# Left atrial expansion index measured with cardiovascular magnetic resonance estimates pulmonary capillary wedge pressure in dilated cardiomyopathy

**DOI:** 10.1186/s12968-023-00977-2

**Published:** 2023-11-30

**Authors:** Davide Genovese, Laura De Michieli, Giacomo Prete, Manuel De Lazzari, Marco Previtero, Donato Mele, Carlo Cernetti, Giuseppe Tarantini, Sabino Iliceto, Martina Perazzolo Marra

**Affiliations:** 1 Cardiology Unit, Department of Cardiac-Thoracic-Vascular Sciences and Public Health, University of Padova, Padova, Italy; 2 Cardiology Unit, Cardio-Neuro-Vascular Department, Ca’ Foncello Hospital, Treviso, Italy

**Keywords:** Left atrial reservoir function, Left atrial compliance, Left atrial volume, Filling pressures, Left atrial pressure

## Abstract

**Background:**

Pulmonary capillary wedge pressure (PCWP) assessment is fundamental for managing dilated cardiomyopathy (DCM) patients. Although cardiovascular magnetic resonance (CMR) has become the gold-standard imaging technique for evaluating cardiac chamber volume and function, PCWP is not routinely assessed with CMR. Therefore, this study aimed to validate the left atrial expansion index (LAEI), a LA reservoir function parameter able to estimate filling pressure with echocardiography, as a novel CMR-measured parameter for non-invasive PCWP estimation in DCM patients.

**Methods:**

We performed a retrospective, single-center, cross-sectional study. We included electively admitted DCM patients referred to our tertiary center for further diagnostic evaluation that underwent a clinically indicated right heart catheterization (RHC) and CMR within 24 h. PCWP invasively measured during RHC was used as the reference. LAEI was calculated from CMR-measured LA maximal and minimal volumes as LAEI =  ( (LAVmax-LAVmin)/LAVmin) × 100.

**Results:**

We enrolled 126 patients (47 ± 14 years; 68% male; PCWP = 17 ± 9.3 mmHg) randomly divided into derivation (n = 92) and validation (n = 34) cohorts with comparable characteristics. In the derivation cohort, the log-transformed (ln) LAEI showed a strong linear correlation with PCWP (r = 0.81, p < 0.001) and remained a strong independent PCWP determinant over clinical and conventional CMR parameters. Moreover, lnLAEI accurately identified PCWP ≥ 15 mmHg (AUC = 0.939, p < 0.001), and the optimal cut-off identified (lnLAEI ≤ 3.85) in the derivation cohort discriminated PCWP ≥ 15 mmHg with 82.4% sensitivity, 88.2% specificity, and 85.3% accuracy in the validation cohort. Finally, the equation PCWP = 52.33- (9.17xlnLAEI) obtained from the derivation cohort predicted PCWP (-0.1 ± 5.7 mmHg) in the validation cohort.

**Conclusions:**

In this cohort of DCM patients, CMR-measured LAEI resulted in a novel and useful parameter for non-invasive PCWP evaluation.

**Supplementary Information:**

The online version contains supplementary material available at 10.1186/s12968-023-00977-2.

## Background

Pulmonary capillary wedge pressure (PCWP) evaluation is fundamental for managing cardiac diseases since PCWP increase is the hemodynamic hallmark of left heart failure syndromes [1]. In clinical practice, PCWP is directly measured during invasive right heart catheterization (RHC). Cardiovascular magnetic resonance (CMR) has become the reference gold-standard imaging technique for evaluating cardiac chambers’ volume and function [2–4]. Small studies demonstrated the feasibility of assessing echocardiographic equivalent diastolic dysfunction parameters with CMR [5–9]. However, despite being promising [10], CMR-based diastolic dysfunction evaluation did not enter widespread clinical practice because it is perceived as cumbersome and impractical; therefore, PCWP evaluation is not performed during routine CMR exams. More recently, Garg. et al. were the first to estimate PCWP using a simple equation that included CMR-measured LA maximal volume (LAVmax) and left ventricular (LV) mass [11]. However, diagnostic performance for identifying elevated PCWP and the agreement with the invasive PCWP measurements were far from optimal [12].

The left atrial expansion index (LAEI) is a simple derived parameter describing left atrial compliance through the relative LA volume increase during the LA reservoir phase. Echo-measured LAEI estimated filling pressures in patients with chronic [13] and acute ischemic heart disease [14], with mitral regurgitation (MR)[15], and in a large cohort of patients with various chronic cardiac diseases [16]. However, whether CMR-measured LAEI could be used for PCWP evaluation has never been previously studied. Therefore, this study aimed to validate, in a cohort of dilated cardiomyopathy (DCM) patients, LAEI as a novel CMR-measured parameter for non-invasive PCWP estimation.

## Methods

### Study population

We performed a retrospective, single-center, cross-sectional study. We screened DCM patients referred for further diagnostic evaluation to our tertiary Center (Department of Cardiac, Thoracic, Vascular Sciences, and Public Health, the University of Padua Hospital) from February 2019 to February 2022. We included only the subject who underwent, within 24 h, clinically indicated RHC and CMR exams. All patients were elective hospitalization, hemodynamically stable, and underwent no therapeutic change between the two exams. We excluded patients with atrial fibrillation (n = 5), patients with MV prosthesis (n = 2), and patients with insufficient CMR image quality related to frequent ventricular ectopic beats such as bigeminy (n = 3).

All enrolled patients were included in the “Padua Cardiac Magnetic Resonance Imaging Registry,” this specific cohort had never been published previously; the local ethics committee approved the study, and all patients provided informed consent. The datasets for the current study are available from the corresponding author upon reasonable request.

### Right heart catheterization

RHC was performed with a Swan-Ganz catheter (SGC) through femoral transvenous access. PCWP values were measured from the pressure–time recordings at the end of a normal expiration by averaging at least three cardiac cycles with the SGC-inflated balloon in the pulmonary capillary wedge position (confirmed by fluoroscopy, pressure-waveform, and oxygen saturation > 95% from a blood sample obtained from the catheter tip). PCWP ≥ 15 mmHg was defined as elevated. [17]

### Cardiovascular magnetic resonance

All patients were imaged using a 1.5T CMR scanner (Magnetom Avanto, Siemens Healthineers, Erlangen, Germany) with an ECG‐triggering and phased array coil system, following the standard protocol [18]. Cine images were acquired during expiratory breath-holds using a balanced, steady-state, free precession (SSFP) and included multiple short-axis (slice thickness 6.0 mm, gap 2.0 mm; repetition time 2.5–3.8 ms; echo time 1.1–1.6 ms, average in-plane resolution 1.5 × 2.4 mm, flip angle 45° to 60°, temporal resolution 40–45 ms) and 4-chambers (ch), 2-ch and 3-ch long axis.

CMR measurements were performed by an operator blinded to RHC and clinical data using CVi42® software (Circle Cardiovascular Imaging Inc, Calgary, Canada). LV and right ventricular (RV) volumes were measured, excluding papillary muscles, from the endocardial border tracings on short-axis images at end-diastole (ED) and end-systole (ES). LV ejection fraction (EF) and RVEF were calculated from the corresponding volumes with the conventional formula. LV mass was calculated by subtracting endocardial from epicardial LV ED volume tracings and multiplying it by 1.05 g/cm^3^. Left atrial maximum volume (LAVmax) and minimum volume (LAVmin) were calculated applying the biplane area-length (BAL) method from the LA areas contoured respectively at ES and ED in both long-axis 4Ch and 2Ch views [2, 4]. Pulmonary veins were excluded from LA tracings. Moreover, also LA appendage was excluded from LA tracings due to its inconsistent visualization in the 2-Ch view. LAEI was calculated using the following formula:

$${\text{LAEI}}\, = \,\left( {\left( {{\text{LAVmax}} - {\text{LAVmin}}} \right)/{\text{LAVmi}}n} \right)\, \times \,100.$$

Furthermore, LV mass and LAVmax were also used for calculating PCWP with the equation proposed by Garg et al. PCWP = 6.1352 +  (0.07204xLAVmax) +  (0.02256xLVmass) [11]. In an independent cohort of 25 patients, the LA volumes and LAEI were measured, in addition to the BAL method, with the short-axis (SAX) volumetry obtained from the LA endocardial border tracings at ED and ES on SSFP short-axis images acquired encompassing the whole left atrium [2, 4].

### Reproducibility analysis

Inter- and intra-reader variability analyses were performed in 20 randomly selected cases with repeated measurements on the same images by the same reader at least four weeks later and by a second independent reader, blinded to all prior measurements.

### Statistical analysis

Continuous variables were summarized as mean ± standard deviation and categorical variables as absolute number with percentage (%). Independent samples T-test and Chi-Square analysis were used for subgroups comparison. Linear correlation was assessed with the Pearson correlation coefficient. lnLAEI was derived by log-transformed LAEI. Multivariate linear regression analysis models tested with the F-test the independent and additive predictive role of lnLAEI for PCWP prediction over clinical and other CMR parameters. Receiver operating characteristic (ROC) curves tested lnLAEI diagnostic accuracy for PCWP ≥ 15 mmHg identification, and the Youden index analysis derived the optimal lnLAEI cut-off. The performance of lnLAEI for elevated PCWP identification was tested and compared against the Garg Eq. with ROC curves analysis in the validation cohort using the De Long method for the area under the curve (AUC) comparisons. The agreement of PCWP = 55.33– (9.17xlnLAEI) with the invasively measured PCWP was analyzed in the validation cohort using Bland–Altman analysis and compared with the performance of Garg Eq.. Inter- and intra-reader variability was tested with the coefficient of variation (CoV) and the intraclass correlation coefficient (ICC). LA volumes and LAEI measured with SAX and BAL methods were compared in an independent cohort of 25 patients using paired T-test and Pearson correlation coefficient analysis. P-value < 0.05 was considered statistically significant. Statistical analysis was performed using SPSS 26.0 (SPSS, Chicago, Illinois, US) and MedCalc 19.6.1 (MedCalc, Ostend, Belgium).

## Results

### Study population

The study population comprised 126 DCM patients (47 ± 14.2 years; 68% male, PCWP = 16.6 ± 9.3 mmHg). Clinical and CMR parameters are summarized in Table 1. We randomly divided our subjects into a derivation (n = 92) and a validation (n = 34) cohort. The clinical and CMR parameters were highly comparable between the two groups (Table 1).

**Table 1:** Study population clinical and CMR parameters and comparison between the derivation and validation cohorts

		Study Population (n = 126)	Derivation (N = 92)	Validation (N = 34)	p
Age (years)		47 ± 14	48 ± 14.4	45 ± 13.6	0.274
Gender (male)		86 (68%)	60 (65%)	26 (76%)	0.230
BMI (Kg/m^2^)		26 ± 3.9	25 ± 3.8	26 ± 4.3	0.618
DCM	* Idiopathic*	75 (60%)	58 (63%)	17 (50%)	0.370
	*Inflammatory*	37 (29%)	24 (26%)	13 (38%)	
	*Other*	14 (11%)	10 (11%)	4 (12%)	
Left bundle branch block		27 (21%)	21 (23%)	6 (18%)	0.530
Systolic blood pressure (mmHg)		115 ± 19	117 ± 20	109 ± 18	0.054
Diastolic blood pressure (mmHg)		72 ± 12	72 ± 11	70 ± 14	0.398
PCWP (mmHg)		17 ± 9.3	17 ± 9.1	17 ± 9.9	0.978
Heart rate (bpm)		75 ± 15	76 ± 16	74 ± 14	0.442
LVEDV (ml/m^2^)		154 ± 45	155 ± 42	151 ± 52	0.706
LVEF (%)		28 ± 11	28 ± 11	29 ± 11	0.529
LV Mass (g/m^2^)		81 ± 28	84 ± 28	75 ± 26	0.143
RVEDV (ml/m^2^)		81 ± 27	80 ± 28	83 ± 27	0.600
RVEF (%)		46 ± 14	46 ± 14	45 ± 14	0.621
LAVmax (ml/m^2^)		53 ± 20	51 ± 18	59 ± 26	**0.031**
LAEI (%)		65 ± 45	66 ± 47	62 ± 41	0.681
lnLAEI		3.90 ± 0.78	3.90 ± 0.80	3.89 ± 0.74	0.927
Mitral regurgitation	*None/trivial*	33 (26%)	23 (25%)	10 (29%)	0.850
	* Mild*	42 (33%)	32 (35%)	10 (29%)	
	* Mild/moderate*	12 (9.5%)	8 (8.7%)	4 (12%)	
	*Moderate*	21 (17%)	15 (16%)	6 (18%)	
	* Moderate/severe*	9 (7.1%)	8 (8.7%)	1 (2.9%)	
	* Severe*	9 (7.1%)	6 (6.5%)	3 (8.8%)	

*BMI* body mass index, *DCM* dilated cardiomyopathy, *PCWP* pulmonary capillary wedge pressure, *LVEDV* left ventricular end-diastolic volume, *LVEF* left ventricular ejection fraction, *RVEDV* right ventricular end-diastolic volume, *LAVmax* left atrial maximal volume, *LAEI* left atrial expansion index, *lnLAEI* log-transformed LAEI

### Derivation cohort

#### LAEI and PCWP correlation

**Fig. 1:**
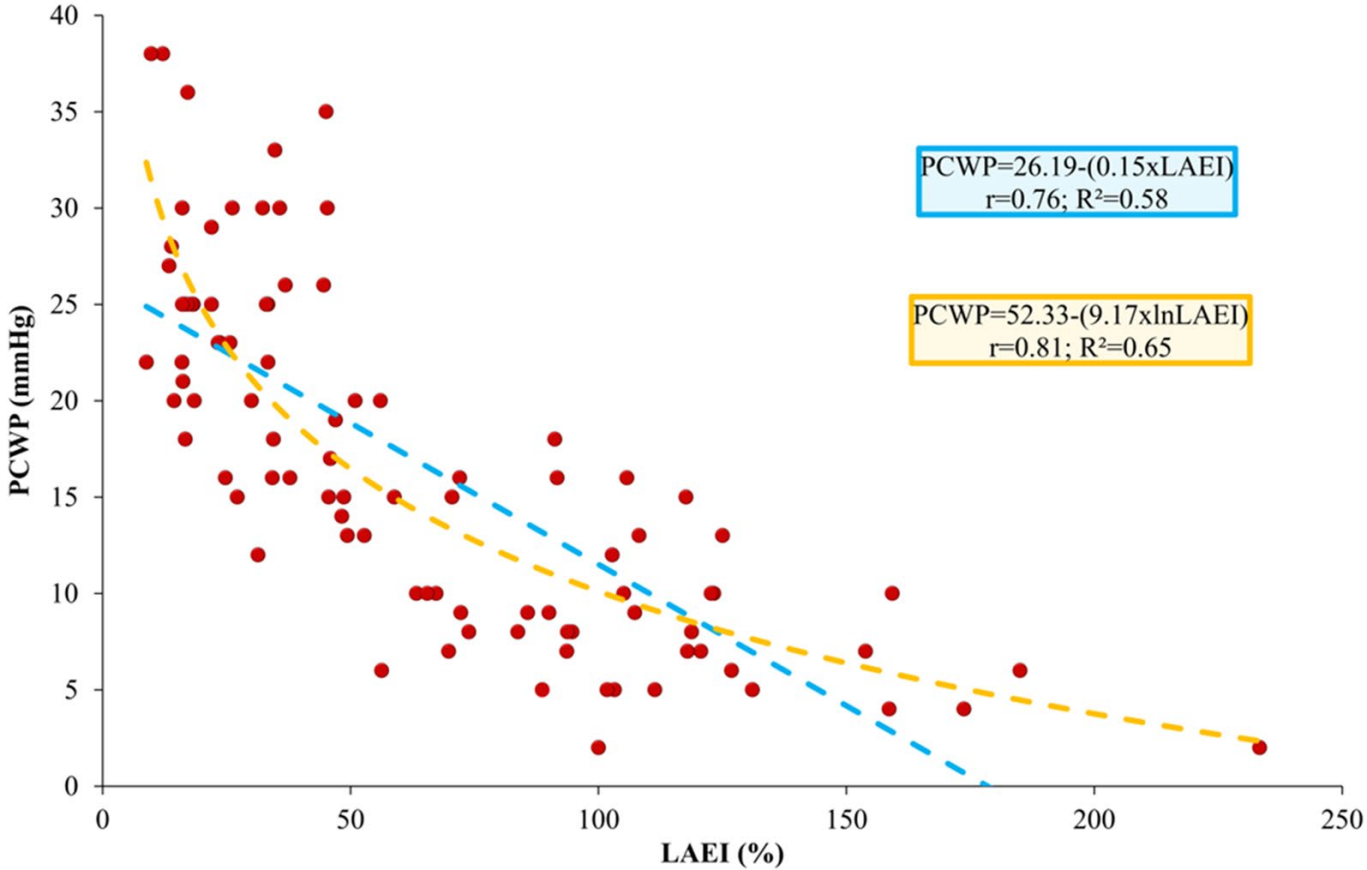
PCWP correlation with LAEI (blue) and lnLAEI (orange) in the derivation cohort

LAEI showed a strong linear correlation with PCWP (r = 0.76; p < 0.001). However, a logarithmic curve better fitted the association between LAEI and PCWP, with a further improvement in lnLAEI linear correlation with PCWP (r = 0.81; p < 0.001) (Fig. 1). PCWP increased, and LAEI decreased with MR worsening (Additional file 1: Table S1), but the logarithmic correlation between PCWP and LAEI was maintained in patients with MR ≥ moderate and with MR < moderate (Additional file 1: Fig. S1).

#### Comparison between elevated and normal PCWP

In the derivation cohort, the subgroup with PCWP ≥ 15 mmHg (n = 52) had a higher heart rate (HR), larger LAVmax, lower systolic blood pressure, LVEF, RVEF, and lnLAEI than the subgroup with PCWP < 15 mmHg (n = 40) (Table 2).

**Table 2:** Clinical and CMR parameters comparison between PCWP ≥ 15 and < 15 mmHg subgroups of the derivation cohort (n = 92)

		PCWP < 15 (n = 40)	PCWP ≥ 15 (n = 52)	p
Age (years)		47 ± 16	49 ± 14	0.462
Gender (male)		24 (60%)	36 (69%)	0.360
BMI (Kg/m^2^)		25 ± 3.5	26 ± 3.9	0.163
DCM	*Idiopathic*	27 (68%)	31 (60%)	0.500
	*Inflammatory*	8 (20%)	16 (31%)	
	*Other*	5 (12%)	5 (9.6%)	
Left bundle branch block		10 (25%)	11 (21%)	0.660
Systolic blood pressure (mmHg)		122 ± 19	113 ± 20	**0.028**
Diastolic blood pressure (mmHg)		72 ± 11	72 ± 12	0.912
PCWP (mmHg)		8.2 ± 3.1	23 ± 6.4	** < 0.001**
Heart Rate (bpm)		68 ± 13	82 ± 15	** < 0.001**
LVEDV (ml/m^2^)		146 ± 40	161 ± 43	0.090
LVEF (%)		34 ± 11	23 ± 8.7	** < 0.001**
LV Mass (g)		84 ± 34	83 ± 23	0.792
RVEDV (ml/m^2^)		75 ± 22	84 ± 32	0.115
RVEF (%)		54 ± 10	40 ± 15	** < 0.010**
LAVmax (ml/m^2^)		46 ± 14	55 ± 19	**0.016**
LAEI (%)		104 ± 41	36 ± 25	** < 0.001**
lnLAEI		4.57 ± 0.41	3.39 ± 0.62	** < 0.001**
Mitral regurgitation	*None/trivial*	12 (30%)	11 (21%)	0.350
	*Mild*	17 (42%)	15 (29%)	
	*Mild/moderate*	2 (5%)	6 (12%)	
	*Moderate*	5 (12%)	10 (19%)	
	*Moderate/severe*	3 (7.5%)	5 (9.6%)	
	*Severe*	1 (2.5%)	5 (9.6%)	

*BMI* body mass index, *DCM* dilated cardiomyopathy, *PCWP* pulmonary capillary wedge pressure, *LVEDV* left ventricular end-diastolic volume, *LVEF* left ventricular ejection fraction, *RVEDV* right ventricular end-diastolic volume, *LAVmax* left atrial maximal volume, *LAEI* left atrial expansion index, *lnLAEI* log-transformed LAEI

#### Univariate and multivariate analysis for PCWP prediction

At the univariate analysis, HR, LVEF, RVEF, LAVmax, MR grade, and lnLAEI resulted in PCWP determinants. The multivariate analysis for PCWP prediction included Model 1 comprising MR grade, LAVmax, RVEF, LVEF, and HR, in which only HR and RVEF remained independent determinants of PCWP. Notably, adding lnLAEI in Model 2 to the variables already included in Model 1 significantly improved the predictive power (Model 1: Adj-R^2^ = 0.422, F = 6.562 vs. Model 2: Adj-R^2^ = 0.682; F = 17.135; p < 0.001 from Model 1). Moreover, lnLAEI remained the only PCWP independent predictor along with HR in Model 2 (Table 3).

**Table 3:** Univariate and multivariate linear analysis for PCWP prediction in the derivation cohort

		r	Univariate			Multivariate					
						Model 1 (Adj-R^2^ 0.422; F = 6.562)			Model 2 (Adj-R^2^ = 0.682; F = 17.135; p < 0.001 from Model 1)		
			Estimate	SE	p	Estimate	SE	p	Estimate	SE	p
Age (years)		0.11	0.07	0.07	0.296						
Gender (male)*			1.633	1.99	0.413						
BMI (Kg/m^2^)		0.12	0.297	0.25	0.240						
DCM**	*Inflammatory*		0.042	2.2	0.985						
	*Other*		− 4.100	3.11	0.190						
Left Bundle Branch Block***			0.008	2.26	0.997						
SBP (mmHg)		0.08	− 0.036	0.05	0.462						
DBP (mmHg)		0.12	0.101	0.09	0.237						
Heart rate (bpm)		0.47	0.270	0.05	** < 0.001**	0.201	0.05	** < 0.001**	0.090	0.04	**0.037**
LVEDV (ml/m^2^)		0.18	0.038	0.02	0.093						
LVEF (%)		0.42	− 0.347	0.08	** < 0.001**	− 0.066	0.09	0.443	0.050	0.07	0.447
LV Mass (g/m^2^)		0.11	− 0.034	0.03	0.319						
RVEDV (ml/m^2^)		0.14	0.046	0.03	0.174						
RVEF (%)		0.49	− 0.308	0.06	** < 0.001**	− 0.158	0.07	**0.026**	0.001	0.06	0.992
LAVmax (ml/m^2^)		0.34	0.176	0.05	** < 0.001**	0.090	0.05	0.074	− 0.039	0.04	0.333
LAEI (%)		0.76	− 0.147	0.01	** < 0.001**						
lnLAEI		0.81	− 9.166	0.71	** < 0.001**				− 9.08	1.12	** < 0.001**
Mitral regurgitation****	* Mild*		2.174	2.38	0.363	1.417	2.00	0.48	0.898	1.49	0.549
	* Mild/moderate*		7.424	3.57	**0.041**	1.476	3.17	0.643	− 0.243	2.38	0.919
	* Moderate*		6.174	2.89	**0.035**	0.962	2.55	0.707	1.281	1.91	0.504
	* Moderate/severe*		8.174	3.57	**0.025**	4.827	3.21	0.137	1.340	2.44	0.584
	*Severe*		9.507	3.99	**0.019**	4.045	3.45	0.244	1.547	2.59	0.552
Intercept						4.363	7.30	0.552	44.909	7.41	** < 0.001**

r: Pearson Coefficient, SE: standard error, Adj-R^2^: Coefficient of determination, F: explained and unexplained variance ratio. Remaining abbreviations as in Table 1. *Female is the reference group; ** Idiopathic is the reference group; *** Narrow QRS is the reference group; ****None/trivial is the reference group

#### ROC curve analysis

**Fig. 2:**
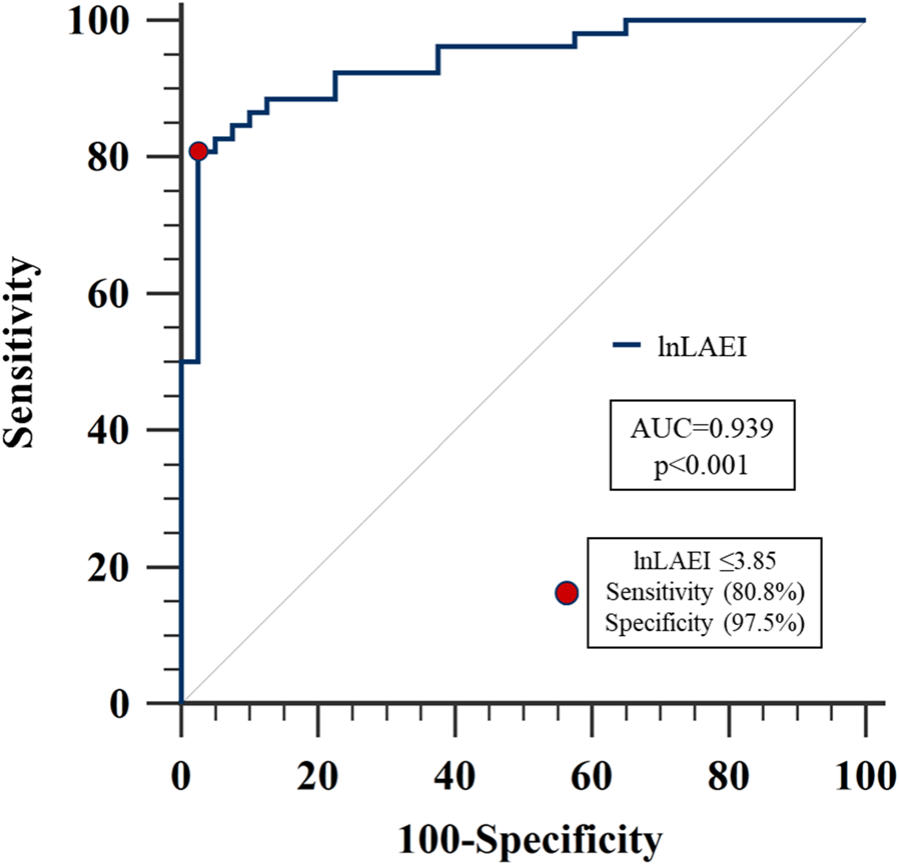
lnLAEI ROC curve for PCWP ≥ 15 mmHg discrimination in the derivation cohort. lnLAEI ≤ 3.85 was the optimal cut-off

lnLAEI identified accurately PCWP ≥ 15 mmHg with an AUC = 0.939 (p < 0.001). The derived optimal cut-off lnLAEI ≤ 3.85 had 80.8% sensitivity and 97.5% specificity for discriminating PCWP ≥ 15 mmHg in the derivation cohort (Fig. 2).

### Validation cohort

#### ROC curve analysis

**Fig. 3:**
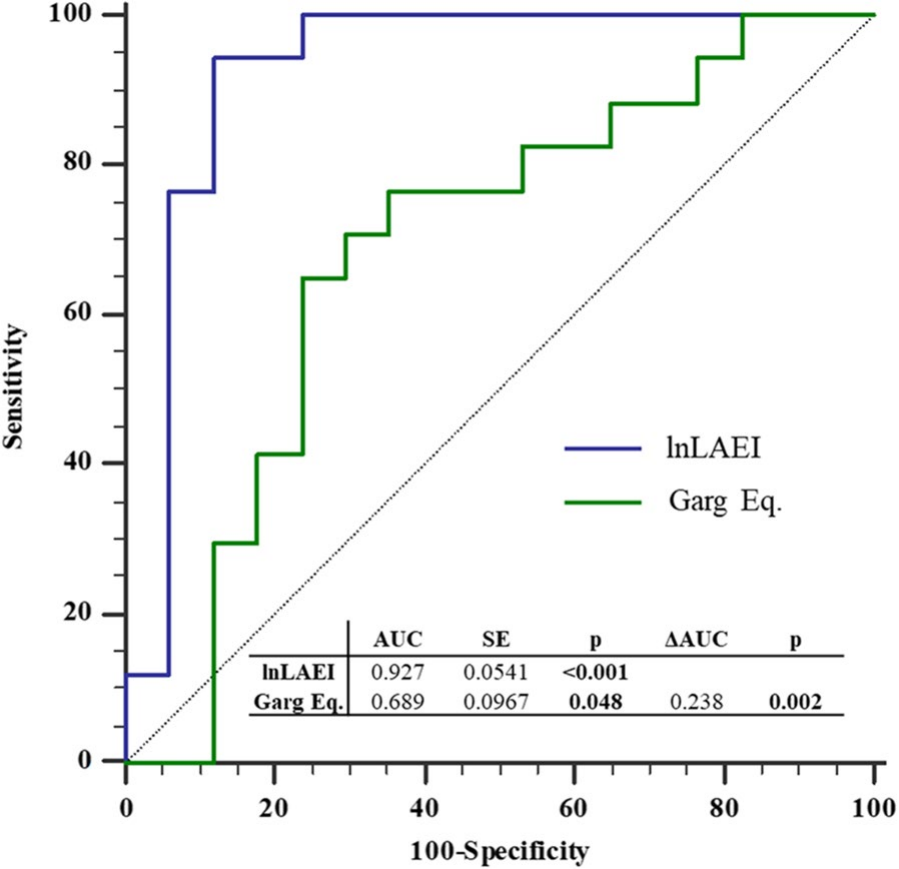
ROC curves comparison between lnLAEI and Garg Eq. for PCWP ≥ 15 mmHg identification in the validation cohort

lnLAEI confirmed an excellent accuracy for PCWP ≥ 15 mmHg identification in the validation cohort (AUC = 0.927, P < 0.001). Furthermore, when lnLAEI AUC was compared with the performance of the Garg Eq. [11] for PCWP ≥ 15 mmHg identification, lnLAEI performed significantly better (ΔAUC = 0.238, p = 0.002) (Fig. 3). The validation cohort optimal cut-off for PCWP ≥ 15 mmHg identification (lnLAEI ≤ 3.89) was superimposable to the lnLAEI cut-off previously identified in the derivation cohort (lnLAEI ≤ 3.85). Moreover, in the validation cohort, lnLAEI ≤ 3.85 had a comparable sensitivity (lnLAEI = 82.4%, Garg Eq. = 88.2%; p = 0.529) but higher specificity (lnLAEI = 88.2%, Garg Eq. = 35.3%; p < 0.001), accuracy (lnLAEI = 85.3%, Garg Eq. = 61.8%; p = 0.041), and positive predictive value (lnLAEI = 87.5%, Garg Eq. = 57.7%; p = 0.010) than Garg Eq. for PCWP ≥ 15 mmHg identification (Table 4).

**Table 4:** Diagnostic accuracy comparison between lnLAEI ≤ 3.85 and Garg Eq. in the validation cohort

	Validation Cohort (n = 34) PCWP ≥ 15 mmHg (n = 17; 50%)		p
	lnLAEI ≤ 3.85 (%)	Garg. Eq (%)	
Sensitivity	82.4	88.2	0.529
Specificity	88.2	35.3	** < 0.001**
Accuracy	85.3	61.8	**0.041**
Positive predictive value	87.5	57.7	**0.010**
Negative predictive value	83.3	75.0	0.433

*BMI* body mass index, *DCM* dilated cardiomyopathy, *PCWP* pulmonary capillary wedge pressure, *LVEDV* left ventricular end-diastolic volume, *LVEF* left ventricular ejection fraction, *RVEDV* right ventricular end-diastolic volume, *LAVmax* left atrial maximal volume, *LAEI* left atrial expansion index, *lnLAEI* log-transformed LAEI

#### lnLAEI equation for PCWP prediction

**Fig. 4:**
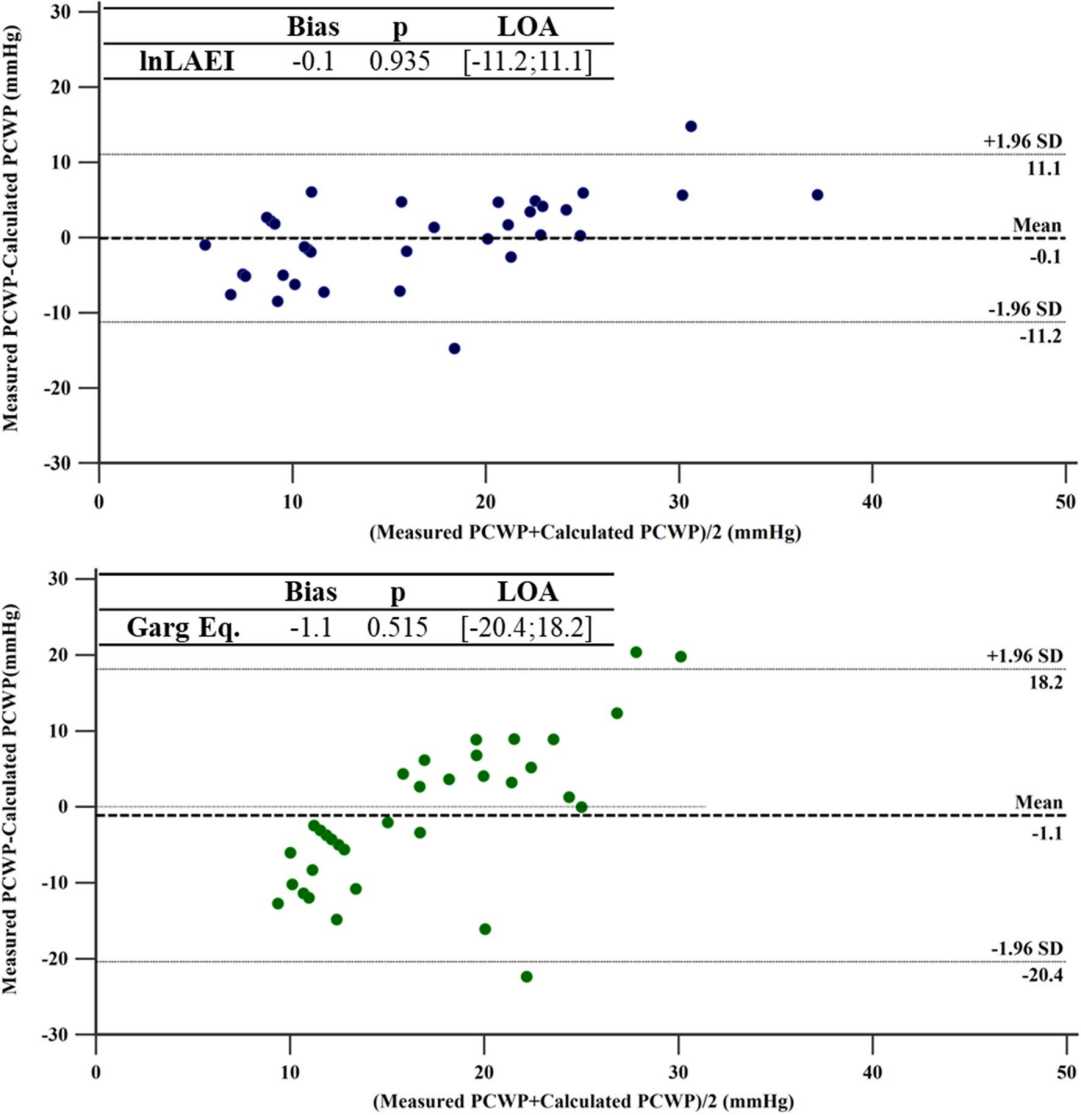
Bland–Altman analysis comparing the invasively measured PCWP with PCWP calculated with lnLAEI Eq. (top) and Garg Eq. (bottom) in the validation cohort

The equation PWCP = 52.33- (9.17xlnLAEI) obtained from the derivation cohort was able to predict in the validation cohort invasively measured PCWP without systematic bias and with a better agreement (bias = 0.1 ± 5.7 mmHg) than Garg Eq. (Bias = -1.1 ± 9.8 mmHg). (Fig. 4).

### Reproducibility analysis

LAEI showed good inter- and intra-reader reproducibility (Table 5).

**Table 5:** Intra-inter reader variability analysis in 20 random cases

	Intra-Reader			Inter-Reader		
	CoV (%)	95%CI	ICC	CoV (%)	95%CI	ICC
LAVmax	0.9	0.6 to 1.2	0.99	2.2	1.4 to 2.9	0.99
LAVmin	1.5	1.0 to 1.9	0.99	1.9	1.3 to 2.6	0.99
LAEI	5.4	3.6 to 7.3	0.98	10.8	7.1 to 14.6	0.97

*CoV* coefficient of variation, *ICC* intraclass correlation coefficient, *CI* confidence interval

### BAL and SAX methods comparison for LA assessment

In an independent cohort of 25 patients, LAVmax, LAVmin and LAEI measured both with BAL and SAX methods were compared. SAX volumetry provided slightly larger LAVmax ( BAL-SAX = − 6.6 ml/m^2^, p < 0.001), LAVmin (BAL-SAX = − 5.4 ml/m^2^, p = 0.002), and slightly lower LAEI values (BAL-SAX = 6.3%, p < 0.002) than the BAL method. Of note, the correlation between the two techniques was excellent for all three parameters (LAVmax = 0.98, LAVmin = 0.99, LAEI = 0.99, all p < 0.001) (Table 6).

**Table 6:** Biplane area-length and short-axis volumetry comparison for LA assessment in an independent cohort of 25 patients

n = 25	BAL	SAX	Correlation analysis		Paired difference analysis	
			r	p	Paired mean difference (BAL – SAX) (ml/m^2^)	p
LAVmax (ml/m^2^)	47 ± 26	53 ± 32	0.98	< 0.001	− 6.6	< 0.001
LAVmin (ml/m^2^)	28 ± 24	33 ± 30	0.99	< 0.001	− 5.4	0.002
LAEI (%)	103 ± 63	97 ± 63	0.99	< 0.001	6.3	0.002

*BAL* Biplane area-length, *SAX* short-axis. Remaining abbreviations as in Table 1

## Discussion

We demonstrated that CMR-measured LAEI provided valuable insight for non-invasive PCWP evaluation in this cohort of DCM patients. The main findings of the study were: (i) LAEI had a strong logarithmic correlation with PCWP; (ii) lnLAEI was an independent determinant of PCWP and provided added predictive value after accounting for other clinical and CMR PCWP determinants; (iii) lnLAEI ≤ 3.85 cut-off obtained from the derivation cohort had 85.3% accuracy in identifying PCWP ≥ 15 mmHg in the validation cohort; (iv) PWCP = 52.33- (9.17xlnLAEI) obtained from the derivation cohort was able to predict PCWP (− 0.1 ± 5.7 mmHg) in the validation cohort; (v) LAEI was more accurate than Garg Eq. in discriminating normal vs. elevated PCWP and for PCWP quantitative estimation.

CMR is the gold standard imaging technique for quantifying the size, mass, and global and regional LV and RV function and accurately assessing myocardial scar and fibrosis. Therefore nowadays, CMR is the reference imaging technique for cardiomyopathies evaluation [19]. However, despite the importance of PCWP as the leading hemodynamic hallmark responsible for heart failure decompensation [20] and its association with outcomes [21, 22], CMR does not currently provide routine non-invasive PCWP evaluation.

In our study, we found that CMR-measured LAEI had a strong logarithmic association with PCWP (r = 0.81; p < 0.001), as previously found with Echo-measured LAEI [13, 16]. Interestingly, CMR-measured lnLAEI identified elevated PCWP in the validation cohort with an accuracy (85.3%) similar to what had been previously found with Echo-measured LAEI (88% in the whole population and 82% in the subgroup with LVEF < 50%)[16] but with an improved intra- inter-operator variability, that resulted comparable to what had been reported in previous CMR studies [23, 24]. In this study, we assessed LA volumes and LAEI with the BAL method, although both BAL and SAX volumetry are currently accepted for LA assessment [2, 4]. We tested the interchangeability of the two approaches in an independent cohort of 25 patients, and we found that both methods provided highly concordant measurements despite slightly larger LA volumes and smaller LAEI values with the SAX volumetry compared to the BAL method. We might speculate that, although we proved the role of LAEI for PCWP assessment solely with the BAL method, SAX volumetry might also be adopted for LAEI calculation and PCWP estimation, although it remains to be formally demonstrated.

Notably, lnLAEI alone explained 65% of the PCWP variance in the derivation cohort in the univariate analysis. In Model 2, R^2^ was only marginally higher (R^2^ = 0.68) than lnLAEI alone (R^2^ = 0.65), underlying the trivial influence of the other parameters on PCWP estimation when lnLAEI was included in the Model. In Model 2, after accounting for clinical and CMR PCWP determinants (HR, LVEF, RVEF, LAVmax and MR), lnLAEI remained the strongest independent determinant of PCWP. Interestingly, despite worse MR determined as expected higher PCWP and lower LAEI, the logarithmic correlation between the two parameters remained stable independently of MR severity, similarly to Echo-measured LAEI [15, 16].

We found lnLAEI to be an accurate parameter for dichotomizing elevated vs. normal PCWP as lnLAEI ≤ 3.85 was able to identify in the validation cohort PCWP ≥ 15 mmHg with an accuracy of 85.3% (sensitivity 82.4%, specificity 88.5%). However, despite the dichotomized approach (normal vs. elevated PCWP) being the current fundament for non-invasive PCWP evaluation as in echocardiography [25], a quantitative estimation of PCWP would be theoretically preferable since PCWP is a continuous parameter. We derived and validated an equation for PCWP estimation from lnLAEI (PWCP = 52.33- (9.17xlnLAEI)) instead of a more complex multivariate regression equation because lnLAEI alone had a predictive power comparable to Model 2 (Model 2: R^2^ = 0.68 vs. lnLAEI: R^2^ = 0.65) with the advantage of providing a practical and user-friendly equation. However, from our findings, the agreement between the lnLAEI equation and invasive PCWP was still modest in some patients, suggesting that the lnLAEI equation should be currently adopted solely as an integrative parameter for further PCWP quantitative insight. Therefore, the dichotomized evaluation of elevated vs. normal PCWP with lnLAEI should currently remain the cornerstone for a reliable non-invasive PCWP assessment with CMR.

Importantly, we found that in our cohort of DCM patients, the CMR-measured LAEI was more accurate than the Garg Eq. for discriminating normal vs. elevated PCWP (Accuracy 85.3% vs. 61.8% for PCWP ≥ 15 mmHg identification) and that PWCP = 52.33- (9.17xlnLAEI) was more accurate than Garg Eq. for quantitative estimation of PCWP. We might speculate that the superiority of lnLAEI over Garg Eq. might be explained by the fact that Garg Eq. was based on sole anatomical parameters (LAVmax and LV mass) and derived from a population that included only 6.2% of patients with reduced LVEF.

Other LA reservoir function parameters might play a role in PCWP assessment, and a recent study showed that LA longitudinal strain assessed during rest and stress CMR discriminated patients with elevated PCWP and heart failure with preserved ejection fraction [26]. These results suggest that CMR-measured LA functional parameters describing the reservoir phase might improve cardiac filling pressure evaluation over static CMR parameters [27], retracing the similar research path recently performed by echocardiography on filling pressure assessment [16, 17, 28–30].

CMR-measured LAEI might become a widespread and practical volume-based LA reservoir parameter for non-invasive PCWP evaluation because it could be easily obtained without additional CMR acquisitions other than conventional cine 4-ch and 2-ch long-axis. Moreover, LAEI calculation is not time-consuming since it only requires the additional measurement of LAVmin to LAVmax, which is already measured in most laboratories. Finally, calculating LAEI does not require any dedicated software package and, therefore, could be promptly integrated into the routine clinical activity of every CMR laboratory.

### Limitations

This study was a single-center and retrospective study. A selection bias is possible since patients were referred to our tertiary center for further diagnostic assessment. However, we focused our research on a selected cohort of patients, and our findings were strengthened by assessing LAEI over a wide range of PCWP values and by providing internal validation of our results in an independent validation cohort. CMR and RHC exams were not simultaneous. However, the time-lapse was minimal, and the patients were hemodynamically stable and did not undergo therapeutic changes between the exams. All patients included were in sinus rhythm; therefore, the performance of LAEI in patients with atrial fibrillation has not been assessed. In this study, LAEI was calculated with the BAL method. Although the SAX volumetry provided highly concordant LAEI values compared to the BAL method in an independent cohort of 25 patients, the two approaches still provided slightly different LA volumes and LAEI values. Therefore, future studies are needed to assess the interchangeability between the approaches for LAEI calculation and PCWP evaluation and other potential differences due to changes in acquisition protocols (i.e., slice thickness and gap).

We did not assess LA strain; therefore, a direct comparison of LAEI with LA strain cannot be performed. Finally, future multicentric and prospective studies are needed to confirm our findings for external validation, to compare different methods for LAEI calculation formally, to explore the performance of LAEI measured with CMR in different cardiac diseases (i.e., heart failure with preserved ejection fraction) and ethnic groups (i.e., different BMI, age).

## Conclusions

In this cohort of DCM patients, CMR-measured LAEI resulted in an accurate parameter for non-invasive dichotomization of normal versus elevated PCWP. Moreover, additional quantitative PCWP insight might be obtained using a simple LAEI-derived equation.

### Supplementary Information

**Additional file 1: Fig. S1.** PCWP and LAEI logarithmic correlation for MR ≥ moderate and MR < moderate. **Table S1.** PCWP and LAEI values by MR degrees.

## Data Availability

The datasets for the current study are available from the corresponding author upon reasonable request.

## Abbreviations

4Ch: Four-chambers
2Ch: Two-chambers
AUC: Area under the curve
BAL: Biplane area-length
CoV: Coefficient of variation
CMR: Cardiovascular magnetic resonance
DCM: Dilated cardiomyopathy
Eq: Equation
ED: End-diastole
ES: End-systole
EF: Ejection fraction
HR: Heart rate
ICC: Intraclass correlation coefficient
LAEI: Left atrial expansion index
lnLAEI: Log-transformed LAEI
LAVmax: Left atrial maximal volume
LAVmin: Left atrial minimal volume
LV: Left ventricular
MR: Mitral regurgitation
MV: Mitral valve
PCWP: Pulmonary capillary wedge pressure
RHC: Right heart catheterization
ROC: Receiver operating characteristic
RV: Right ventricular
SGC: Swan-Ganz catheter
SAX: Short-Axis
