# Supplementary material for: Left atrial expansion index measured with cardiovascular magnetic resonance estimates pulmonary capillary wedge pressure in dilated cardiomyopathy
Source: J Cardiovasc Magn Reson. 2023 Nov 30;25:71. doi: 10.1186/s12968-023-00977-2 (PMC10688459; doi:10.1186/s12968-023-00977-2)
Supplement: Supplementary file 1 — Additional file 1: Fig. S1. PCWP and LAEI logarithmic correlation for MR ≥ moderate and MR < moderate. Table S1. PCWP and LAEI values by MR degrees. [file 12968_2023_977_MOESM1_ESM.docx]

**ADDITIONAL MATERIAL**

**Figure S1.** PCWP and LAEI logarithmic correlation for MR≥moderate and MR<moderate.


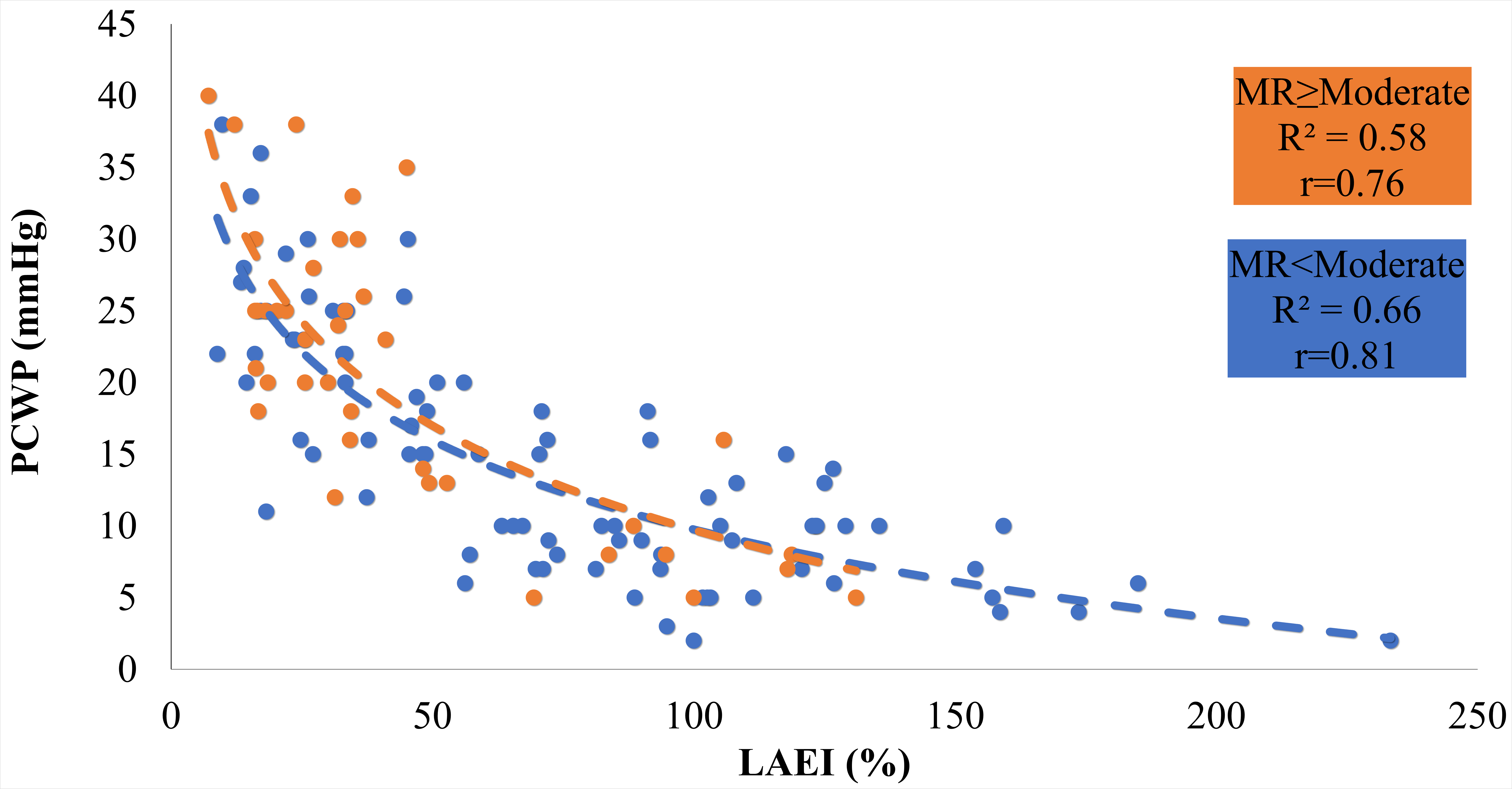


**Table S1**. PCWP and LAEI values by MR degrees.

| **MR** | **PCWP (mmHg)** | | **LAEI (%)** | |
| --- | --- | --- | --- | --- |
| **None/trivial (n=33)** | 13 | ±8.2 | 84 | ±53 |
| **Mild (n=42)** | 15 | ±8.5 | 71 | ±40 |
| **Mild/Moderate (n=12)** | 18 | ±8.1 | 52 | ±48 |
| **Moderate (n=21)** | 19 | ±10.2 | 51 | ±35 |
| **Moderate/Severe (n=9)** | 22 | ±10.2 | 40 | ±36 |
| **Severe (n=9)** | 24 | ±7.9 | 37 | ±32 |
|  |  |  |  |  |

**Abbreviations.** As in Table 1
